# Abdominal and Pelvic Pain: Current Challenges and Future Opportunities

**DOI:** 10.3389/fpain.2021.634804

**Published:** 2021-02-04

**Authors:** Tian Yuan, Beverley Greenwood-Van Meerveld

**Affiliations:** ^1^Department of Physiology, University of Oklahoma Health Sciences Center, Oklahoma City, OK, United States; ^2^VA Health Care System, Oklahoma City, OK, United States

**Keywords:** visceral pain, analgesic, sex, anxiety and depression, stress, epigenetics, peripheral sensitization, central sensitization

## Introduction: The Burden of Visceral Pain

Visceral pain describes pain emanating from internal thoracic, pelvic, or abdominal organs. Although visceral pain is common and is a leading worldwide cause of healthcare utilization [1, 2], the classification of chronic visceral pain is complex, often resulting from persistent inflammation, vascular (ischemic) mechanisms, cancer, obstruction, luminal distension, traction, or compression, as well as unexplained functional mechanisms. Chronic visceral pain is the hallmark feature of many disorders, some with distinct organ pathology, including pancreatitis, inflammatory bowel disease (IBD), interstitial cystitis (IC)/painful bladder syndrome (PBS), and gynecological pain. In other visceral pain disorders such as irritable bowel syndrome (IBS) and functional dyspepsia (FD) there is no evidence of any structural or histological abnormalities to explain the pain. Interestingly, chronic visceral pain is more common in women, who tend to have lower thresholds and less tolerance to nociceptive stimuli whereas no sex differences in visceral sensitivity are observed in healthy controls [3–7]. When asked to describe their experience of abdominal or pelvic pain, a challenge is that patients will find this difficult and often describe their pain is dull and diffuse. In addition, patients often incorrectly localize the origin of their visceral pain. When clinicians are confronted with patients suffering from visceral pain, another challenge arises because the characteristics of visceral pain severely hinder their ability to make the correct diagnosis and initiate an effective treatment approach. All too often patients with chronic visceral pain are dismissed and as a consequence, patients will seek support from multiple physicians, tolerate many doctor visits, and endure multiple treatment approaches before the origin of the visceral pain is correctly diagnosed and managed. Previous episodes of infection/inflammation and early life adversity serve as risk factors for the development of abdominal and pelvic pain [8–13]. Moreover, we know that exposure to persistent mental or social stress can trigger, exacerbate, and maintain visceral pain [8, 14, 15].

The mechanisms that control and modulate chronic pain are poorly understood; visceral pain may arise due to peripheral augmentation of primary afferent signals as well as central facilitation at spinal and supraspinal levels affecting descending nociceptive modulation. In support, a history of early life adversity, as well as adult stress and anxiety often exacerbate visceral pain. Multiple visceral pain disorders are often comorbid with other mood and anxiety disorders. Furthermore, due to peripheral and central cross-sensitization, pain in one organ can lead to the perception of pain in an adjacent visceral organ (e.g., bladder and colon). Taken together, there is a huge medical need to understand the underlying the mechanisms responsible for visceral pain in order to aid in the development of better treatment approaches that may lead to the future development of novel compounds to treat chronic abdominal and pelvic pain.

## Current Challenges and Opportunities

### Few Therapeutic Options to Treat Visceral Pain

Acute visceral pain can be treated with classical analgesics and the transient pain is ultimately subdued by treating the underlying cause, which often involves attenuating the inflammatory response in the visceral organ. However, for a significant number of patients, visceral pain is chronic and often debilitating. The current clinical management options for chronic visceral pain remain inadequate and providing relief for patients with abdominal and pelvic pain remains a considerable clinical challenge. In the past, opioids were the first-line treatment approach for chronic and severe visceral pain, but this approach is limited by a lack of efficacy, high abuse potential, and significant side-effects such as drowsiness, constipation, and nausea. Moreover, long term opioids use can paradoxically lead to a visceral hyperalgesia and thus worsening of pain rather than having a beneficial effect. Clinical evidence now suggests that severe or refractory abdominal pain, associated with IBS, can be managed though the judicious use of neuromodulators such as tricyclic antidepressants, including amitriptyline and nortriptyline, given at doses below the psychiatric range [16]. Although symptoms and comorbidities of visceral pain disorders overlap, there is no “single” treatment for these disorders. Another significant challenge is the lack of definitive biomarkers for visceral pain. Although preclinical studies in animal models of visceral hypersensitivity have identified multiple potential therapeutic targets, a more sustained effort supporting basic research into the physiology and pathophysiology of visceral pain will provide us with opportunities to identify novel targets for future drug development. We also believe that transitioning to personalized medicine and targeted therapeutic interventions will likely be an essential prerequisite for the successful treatment of abdominal and pelvic pain. But before we reach the goal of managing visceral pain and thus allowing patients to lead productive and pain-free lives, we still have a rather long and likely a winding path to travel.

### Pathophysiology of Visceral Pain Is Incompletely Understood

The causes of chronic visceral pain are incompletely understood due to the multifactorial etiology of the disorder. Chronic visceral pain is a classic example of gene × environment interaction which results from maladaptive changes in neuronal circuitry leading to plasticity of aberrant signals. Pathological sensitization of primary afferents in the visceral organ can occur due to multiple mechanisms. In the *peripheral organ*, crosstalk between immune cells, nerves, or the gut microbiota, can enhance nociceptive signaling and even lead to activation of “silent” nociceptors. In the *spinal cord*, enhanced pain signals can sensitize the dorsal horn neurons [17, 18]. There can also be an attenuation of endogenous descending inhibitory signaling from the brain to dampen the nociceptive signal [19–22]. Within *supraspinal structures*, exposure to chronic stress can remodel neural circuits that promote central sensitization and can inhibit descending inhibition.

A future opportunity that may allow for us to develop a better understanding of chronic visceral pain may arise from research in field of epigenetics. A potential mechanism underlying the persistent effects of stress on visceral sensitivity could be epigenetic modulation of gene expression in the periphery, spinal cord, and brain. Epigenetic mechanisms stably reprogram gene expression and likely embed time-limited exposures into long-term gene expression programs that “memorize” the initial encounter [23, 24]. While there are a limited number of reports examining epigenetically mediated mechanisms involved in visceral nociception, stress-induced visceral pain has been linked to alterations in DNA methylation and histone acetylation patterns within the brain and spinal cord, leading to increased expression of pro-nociceptive neurotransmitters [25–30]. Although exploring this concept remains in its infancy future research may provide an explanation for why patients suffer from chronic visceral pain after an initial sensitizing event such as an acute inflammation or following exposure to early life adversity.

### Sex as a Biological Variable in Visceral Pain Disorders

Although there is a growing body of evidence reporting a female-predominance in the prevalence of chronic visceral pain, the molecular mechanisms of pain vulnerability in females are poorly understood but delineating these is an exciting future research opportunity. Studying the epigenome in the context of sex differences, at the most basic level, may reveal that these can occur via programming of genes to be more vulnerable or resilient to manipulation in the presence of the sex hormones estrogen or testosterone. There are also opportunities to build upon evidence suggesting visceral pain vulnerability may be due to sex-differences in CNS processing of visceral information. In support, positron emission tomography (PET) imaging indicates there are sex-related differences in regional brain responses to provocative stimuli in patients with IBS [31].

### Visceral Pain and Overlapping Comorbidities

Visceral pain is frequently co-morbid with mood disorders such as anxiety and depression [32–34]. A further characteristic of pain in IBS is that it is associated with cross-organ sensitization of other viscera. As many as 30–50% of patients diagnosed with IBS exhibit symptoms of overactive bladder and painful bladder syndrome (PBS), while a large cohort of patients diagnosed with PBS exhibit symptoms that fulfill the criteria for IBS [35–40]. Furthermore, dysmenorrhea of uterine origin can result in colonic allodynia to physiological stimuli such as the presence of gas of fecal material in the colon [41]. Although the mechanisms by which visceral pain overlaps with other comorbidities remain to be further elucidated, it is clear that persistent stress exaggerates pain perception and sensitizes pain pathways, leading to a feed-forward cycle promoting chronic visceral pain disorders.

### Challenges With Experimental Models of Visceral Pain

Considering that chronic visceral pain is a major driving predictor of healthcare seeking behaviors and poor work productivity, the development of novel therapeutic agents, based on validated animal models and assays, is central to improving outcomes in the patients. Multiple challenges exist in using animal models to further understand the pathophysiology and treatment of visceral pain. One key challenge we face is that no single experimental model recapitulates all aspects of abdominal and pelvic pain based upon the multifactorial nature of visceral pain disorders and their complicated interactions and overlap with other visceral pain disorders and with mood disorders such as anxiety and depression. Another challenge is that visceral pain disorders are predominantly observed in women and animal models must recapitulate this clinical reality. Taken together these challenges make visceral pain disorders difficult to reproduce in experimental models. However, the future use of the latest biological and neuroimaging tools in animal studies will enhance our basic understanding of visceral pain that will likely lead to the identification of validated biomarkers that will produce better diagnoses and treatment approaches for patients with severe abdominal and pelvic pain. While all currently approved drugs for visceral pain have been shown to be efficacious in animal models of visceral hypersensitivity, a conundrum is that not all drugs that are effective in animal models have proven to be clinically effective. Furthermore, it is unclear why when a large number of targets have shown efficacy in animal models of visceral hypersensitivity, have not led to so few therapeutic options for patients. However, as newer therapies become available for clinical use based upon data obtained in relevant animal models, future research should encourage the use of the same models for reverse translation i.e., from the clinic back to the animal model to further refine the novel therapeutic approaches.

## Summary and Conclusions

The goal of this new journal and more specifically, the specialty section on *Abdominal and Pelvic Pain* is to advance research and knowledge surrounding the underlying central neurocircuitry of visceral pain, in an attempt to identify and then validate molecular targets for novel analgesics. Research efforts will focus on characterizing the mechanisms of visceral pain, including abdominal pain, chronic pelvic pain (CPP) and chronic pelvic pain syndrome (CPPS); debilitating conditions that present a public health crisis and a burden on healthcare expenditure. Examples of visceral pain syndromes include Vulvar Pain Syndrome (Vulvodynia), Vestibular Pain Syndrome, Clitoral Pain Syndrome, Dysmenorrhea, Endometriosis-Associated Pain Syndrome, Irritable Bowel Syndrome and Pelvic Floor Muscle-pain Syndrome. This section will encourage the latest, high-quality, cutting edge research focused on identifying and elucidating the role of central and peripheral neuroplasticity in visceral pain syndromes. Areas that will be covered by this section, include but are not limited to neurogastroenterology and the enteric nervous system, the role of the CNS in pathogenesis of chronic visceral pain, stress-induced visceral hypersensitivity; the molecular mechanisms underlying sex-related differences in visceral pain and pain vulnerability vs. resilience, novel and promising approaches in the development and treatment for visceral pain.

## Author Contributions

All authors participated in the inception, writing, commenting of this editorial, and approved the paper.

## Conflict of Interest

The authors declare that the research was conducted in the absence of any commercial or financial relationships that could be construed as a potential conflict of interest.
